# A comparative assessment of prostate positioning guided by three-dimensional ultrasound and cone beam CT

**DOI:** 10.1186/s13014-015-0380-1

**Published:** 2015-04-09

**Authors:** Minglun Li, Hendrik Ballhausen, Nina-Sophie Hegemann, Ute Ganswindt, Farkhad Manapov, Stefan Tritschler, Alexander Roosen, Christian Gratzke, Michael Reiner, Claus Belka

**Affiliations:** Department of Radiation Oncology, University Hospital Munich, Ludwig-Maximilians-University, Munich, Germany; Department of Urology, University Hospital Munich, Ludwig-Maximilians-University, Munich, Germany

**Keywords:** Prostate cancer, Radiotherapy, Ultrasound, Setup error, Patient positioning, IGRT, CBCT, 3DUS, Clarity

## Abstract

**Background:**

The accuracy of the Elekta Clarity™ three-dimensional ultrasound system (3DUS) was assessed for prostate positioning and compared to seed- and bone-based positioning in kilo-voltage cone-beam computed tomography (CBCT) during a definitive radiotherapy.

**Methods:**

The prostate positioning of 6 patients, with fiducial markers implanted into the prostate, was controlled by 3DUS and CBCT. In total, 78 ultrasound scans were performed trans-abdominally and compared to bone-matches and seed-matches in CBCT scans. Setup errors detected by the different modalities were compared. Systematic and random errors were analysed, and optimal setup margins were calculated.

**Results:**

The discrepancy between 3DUS and seed-match in CBCT was −0.2 ± 2.7 mm laterally, −1.9 ± 2.3 mm longitudinally and 0.0 ± 3.0 mm vertically and significant only in longitudinal direction. Using seed-match as reference, systematic errors of 3DUS were 1.3 mm laterally, 0.8 mm longitudinally and 1.4 mm vertically, and random errors were 2.5 mm laterally, 2.3 mm longitudinally, and 2.7 mm vertically. No significant difference could be detected for 3DUS in comparison to bone-match in CBCT.

**Conclusions:**

3DUS is feasible for image guidance for patients with prostate cancer and appears comparable to CBCT based image guidance in the retrospective study. While 3DUS offers some distinct advantages such as no need of invasive fiducial implantation and avoidance of extra radiation, its disadvantages include the operator dependence of the technique and dependence on sufficient bladder filling. Further study of 3DUS for image guidance in a large patient cohort is warranted.

## Introduction

External beam radiotherapy (EBRT) is a mainstay for curative therapy of localized prostate cancer [1-3]. A dose–response relationship has been clearly addressed in several randomized clinical trials [4-6]. Recent technologic advances in radiotherapy allow a better dose conformality around target volumes and an adequate sparing of normal tissues, which facilitates a dose escalation to improve clinical outcomes without a relevant increase of side effects [5,7-9]. However, due to reduced margins, the precise application of radiotherapy has become more sensitive to geometric uncertainties [10,11]. In the case of prostate cancer, these uncertainties are mainly introduced by organ motion [12]. The movement of the prostate between fractions (inter-fraction motion) dominates setup errors and mainly contributes to the deviation of dose distribution to the target volumes and normal tissues [13,14], before and in addition to the movement during a fraction (intra-fraction motion) [15-18].

Depending on the filling of rectum and bladder, the inter-fractional movement of the prostate in the pelvis can amount to more than 1 cm [19-22]. A precise dose application to the target volume is only possible after a precise repositioning of the prostate via image guidance (Image-guided radiotherapy, IGRT) before each fraction. For IGRT, prostate motion can be detected and measured with several methods, including cone beam computed tomography (CBCT) and portal images with implanted radiopaque fiducials [23-25], ultrasound (US) [23,26-28] and the Calypso system [29,30]. Fiducial-based image guidance is the most widely accepted method, considered as a gold standard [24], while 3DUS-based technique has its distinct advantages such as non-invasiveness and superior soft tissue contrast.

The Clarity™ localization and positioning system is the latest generation of US-based guidance systems from Elekta Company, using three-dimensional image data and offers a significant improvement in image quality over earlier systems. Unlike earlier inter-modality systems such as B-mode Acquisition and Targeting System (BAT®), SonArray®, Clarity offers a true intra-modality verification method [31,32]. For this purpose, a simulation 3DUS scan is acquired trans-abdominally in the planning phase. Before each fraction of radiotherapy, a 3DUS is obtained and matched to the simulation 3DUS, providing online correction of the prostate position. Such an intra-modality method has been proven to offer improved accuracy for prostate alignment over cross-modality method which compares treatment US to planning CT [27].

To date, some data and clinical experience with Clarity 3DUS has been reported [26]. In this manuscript we quantify the accuracy of stereotactic Clarity 3DUS unit using a simulation CT/kilovolt-CBCT system as counterpart for daily prostate repositioning during radiotherapy in patients with implanted gold marker fiducials.

## Patients and methods

### Patients and treatment course

Setup errors of six prostate cancer patients during definitive EBRT were retrospectively evaluated. These patients had each received three gold markers implanted in the urological department in domo under trans-rectal ultrasound guidance around two weeks before the simulation CT (details see [33]). All patients were advised to follow a protocol to ensure a moderately filled bladder and an empty rectum before planning CT and every day before radiotherapy. In case of a flatulent rectum or an empty bladder, patients received enema to empty their rectum or drank water to fill their bladder, and 3DUS and CT scans were repeated if possible with the agreement of patients.

Patients were treated with a 6-MV linear accelerator (Elekta Synergy) with intensity-modulated radiotherapy (IMRT) plans. Positioning was performed by one of several trained users (two radiation oncologists and three radiotherapy technologists, all with user training courses for CBCT and 3DUS). All patients were aligned to skin marks before treatment. The remaining inter-fractional setup error was controlled by 3DUS whenever possible. About twice a week, CBCTs were performed for bone-match and seed-match.

### 3DUS acquisition and quality assurance

**Figure 1:**
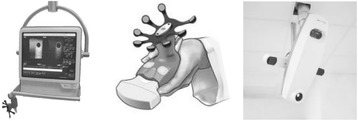
**Elekta Clarity** **™** **system for transabdominal 3D ultrasound: mobile bedside workstation (left), free-hand probe with infrared reflectors (middle), and ceiling-mounted stereoscopic infrared camera (right, two arrows).**

The 3DUS system consisted of two identical Clarity units, one in the room of planning CT, and one in the treatment room. Each Clarity unit consists of a mobile US work-station with touch-screen and a free-hand US probe with 8 infrared reflectors firmly installed on it (Figure 1). Two ceiling-mounted stereoscopic infrared cameras track the US probe and calculates the geometric positions of the scanned structures in US images. For scanning, the US probe was manually placed 5–10 cm supra-pubic on the abdomen with a moderate pressure for a good image quality. Then the US probe is swept from superior to inferior, without translatory movement of the probe, to scan the prostate and bladder from retropubic to the top of bladder. If necessary, the placing position of US probe on the abdomen was changed for a possible complete vision of the bladder and prostate. After that the US probe was removed from patient. Based upon the primary US data, its 3D imaging was secondarily generated and presented in the workstation.

For quality assurance, the Clarity system had been calibrated to its inherent technical limit of about 1 mm radially, using a calibration phantom [34]. The Clarity positioning is based on soft tissue anatomy since gold markers are not clearly visible in US images. All 3DUS were retrospectively revised by the physicians (ML, FM). No rotational errors were calculated in Clarity system. Setup errors were represented in the same coordinate system as those reported by CBCT.

### Simulation

During simulation, after definition of the reference point and application of skin markers, a reference US scan was acquired trans-abdominally with the patient in his later treatment position. Directly after that, the US probe was removed and a simulation CT for treatment planning was acquired as a sequential scan with a slice thickness of 3 mm (Aquilion LB, Toshiba, Japan). The delay between 3DUS and CT scan was kept as brief as possible, to reduce the risk of involuntary patient motion.

**Figure 2:**
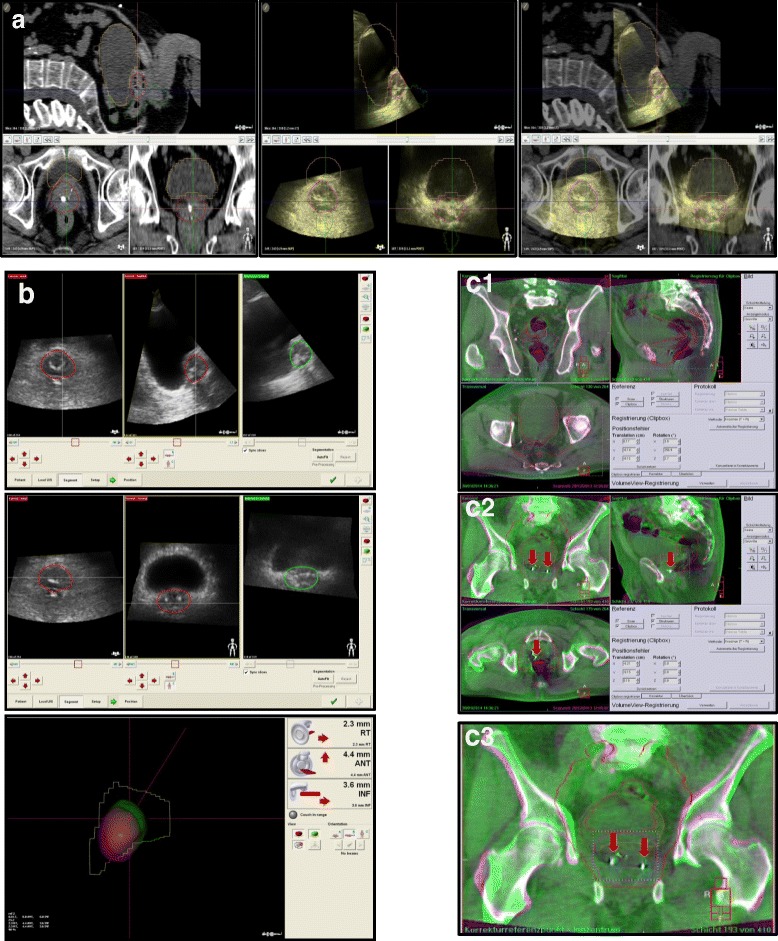
**Example images for the workflow of Clarity 3DUS system and bone-/seed-match in CBCT. a)** Automatic fusion of a 3DUS scan to a simulation CT in the planning phase. **b)** On-line alignment of Prostate organ in Clarity system during the treatment phase. **c)** Bone-Match (c1) and Seed-Match (c2, c3) with zoom-in view on the axial plane in CBCT. Red arrows indicate the implanted seeds in the prostate.

After the planning process, the radiotherapy plan, complete with CT datasets and all contours including Clinical target volume (CTV), Planning target volume (PTV) and Organs at risk (OAR), was imported into the Clarity planning workstation and registered to the US reference scan (Figure 2a). The contour of the prostate, based on ultrasound images, was drawn and added as the Positioning reference volume (PRV) for US-based positioning during the treatment.

All images of simulation CT and all contours in DICOM format were imported to the XVI control workstation (XVI software version 3.5; Elekta) as reference for CBCT scans later.

### CBCT acquisition

CBCT provided volumetric data sets for on-line correction. The X-ray source was operated at nominal levels suggested by the manufacturer (120 kV, 25 mA, and 40 ms). An extensive quality assurance program was in place to assess image resolution, image distortion, and precision of isocenter detection routinely on a weekly basis. The projections were processed to 3D volume images with Elekta’s XVI software. CBCT images were on-line matched to simulation CT images, using automatic “bone fusion” or “seed fusion” mode in the XVI software.

### Repositioning protocol and image analysis

For the patients with implanted gold markers, the following imaging protocol was sequentially performed: after initial positioning of skin marks to room lasers, a 3DUS scan was performed and the setup errors were documented without correction (Figure 2b). Whenever a CBCT scan was performed, it was performed right after the 3DUS scan to minimize patient motion in-between these two measurements.

Next, the XVI program was employed to match bony structures based on a clip box containing all pelvic bony structures (Figure 2c1; clip box positioned on planning CT). Again, the setup errors were documented without correction. Finally, a seed-match using a small alignment clip box containing only the prostate and the seeds was performed. The actual correction before application of this fraction of radiotherapy was then based on the setup errors according to implanted seeds (Figure 2c2 and 2c3).

### Data analysis and statistics

Fiducial-based alignment is the classical und widespread technique for prostate positioning [24], thus its setup errors were used as reference to evaluate the accuracy of 3DUS alignments, bone-match in CBCT and skin marks. Hence, the mean, standard deviation, median and range of residual setup errors of 3DUS, bone-match in CBCT and skin marks, as the difference to seed-match in CBCT, were calculated for each axis across all fractions. Significance with respect to seed-match in CBCT was then evaluated by the two-sided Student’s t-test in comparison to the hypothetical zero mean. Significance of the differences among 3DUS, bone match in CBCT and skin marks was tested by the paired two-sided Student’s t-test.

The mean and standard deviation were also calculated for every patient individually and the respective systematic error (inter-patient variability) and random error (intra-patient variance) were calculated as below:

Setup error in patient **j** during fraction **i**: *d*_*ij*_

Define the average ${m}_j=\overline{d_{ij}}$

And the variance ${v}_j=\overline{\left({d}_{ij}-{m}_j\right)2}$

The systematic error is ${\displaystyle \sum =\sqrt{\overline{\left({m}_j-\overline{m_j}\right)2}}}$

The random error is $\sigma =\sqrt{\overline{v_j}}$

These errors were translated into the respective CTV to PTV margins using the optimal margin recipe by van Herk [35]. The confidence interval of the systematic error was calculated based on the appropriate percentile of the χ^2^ distribution. The limits of the confidence interval of the random error were estimated as the roots of limits of the confidence interval of the appropriate quantile of the Student’s t-distribution for the variance. The confidence interval of the resulting margin was conservatively estimated from the combined lower resp. upper limits of the confidence intervals of the random and systematic error.

## Results

### Viability of 3DUS and availability of comparisons

Patients were aligned to skin marks before all of the 183 treatment sessions. Control of the remaining inter-fractional setup error by 3DUS was successfully employed 154 times (84%). During the remainder of fractions, insufficient bladder filling (~8% of fractions) and patient movement (~5% of fractions) were the most frequent obstacles to 3DUS. CBCTs were performed for a total of 90 sessions (49%). Out of these 154 resp. 90 sessions, simultaneous 3DUS and CBCT was available for 78 sessions (43%). The following analysis is based on the data from these 78 fractions, and results are presented relative to the reference value provided by seed-match in CBCT.

### Comparison of setup errors from 3DUS vs. seed-match in CBCT

The differences of prostate positioning between 3DUS and seed-match in CBCT ranged from −5.6 to 6.9 mm in the lateral direction, from −10.0 to 2.9 mm in the longitudinal direction, and from −7.3 to 7.2 mm in the vertical direction. The average discrepancies ± SD were −0.2 ± 2.7 mm, −1.9 ± 2.3 mm and 0.0 ± 3.0 mm in the lateral, longitudinal and vertical directions, respectively (Table 1). In the lateral (t = 0.52, p = 0.60) and vertical direction (t = 0.14, p = 0.89) the discrepancies were consistent with zero (by Student's two-sided one-sample t-test for N = 78 minus one degrees of freedom). The discrepancy in longitudinal direction, however, was significantly different from zero (t = 7.35, p < 0.001).

**Table 1:** **Setup errors as detected by 3DUS, CBCT, and skin marks**

		**Mean ± SD**	**Median**	**Range**	**≤5 mm**
**Lateral axis**					
	3DUS	−0.2 ± 2.7	−0.5	−5.6 … 6.9	92%
	Bone-match	−0.3 ± 1.3	−0.3	−3.7 … 1.7	100%
	Skin marks	−0.4 ± 2.6	−0.3	−6.1 … 4.5	95%
**Longitudinal axis**					
	3DUS	−1.9 ± 2.3	−2.0	−10.0 … 2.9	94%
	Bone-match	−2.1 ± 2.5	−2.3	−7.5 … 8.9	90%
	Skin marks	−1.5 ± 3.5	−1.6	−8.4 … 7.5	78%
**Vertical axis**					
	3DUS	0.0 ± 3.0	0.2	−7.3 … 7.2	92%
	Bone-match	0.2 ± 3.0	−0.3	−5.3 … 9.4	92%
	Skin marks	−0.8 ± 4.3	−0.8	−10.6 … 7.5	71%

In units of mm; relative to position readings by seed-match; N = 78.

Inter-patient variability was substantial, with the average discrepancy varying between −1.8 mm and +1.7 mm in the lateral direction, between −3.3 and −0.9 mm in the longitudinal direction, and between −2.1 and +2.0 mm in the vertical direction.

77%, 92%, and 100% of discrepancies were less than 3, 5, and 10 mm laterally, respectively. 71%, 94%, and 100% of discrepancies were less than 3, 5, and 10 mm longitudinally, respectively. 60%, 92%, and 100% of discrepancies were less than 3, 5, and 10 mm vertically, respectively.

The absolute value of the differences of prostate positioning between 3DUS and seed-match in CBCT amounted to up to 11 mm in the radial direction. The average absolute discrepancies ± SD were 2.1 ± 1.6 mm, 2.4 ± 1.8 mm and 2.4 ± 1.8 mm, in the lateral, longitudinal and vertical directions, respectively.

### Comparison of accuracy of 3DUS to bone-match in CBCT and to skin marks

In full analogy, also the positioning errors as detected by bone-match in CBCT were compared to the reference values provided by seed-match in CBCT. Similarly, the residual positioning errors after initial positioning to skin marks were detected by seed-match in CBCT. As above, see Table 1.

By the two-sided paired Student’s t-test, there was no significant difference between 3DUS and bone-match in CBCT (p = 0.52, p = 0.74 and p = 0.71 laterally, longitudinally, and vertically, respectively). Neither was there any significant difference between 3DUS and skin marks (p = 0.53, p = 0.36 and p = 0.10 laterally, longitudinally and vertically, respectively).

Among the other three modalities (seed-match, bone-match and skin marks), there were significant differences between bone-match and seed-match in CBCT in the lateral (p = 0.019) and longitudinal (p < 0.001) directions, and between skin-marks and bone-match in CBCT in the vertical direction (p = 0.004).

### Systematic and random errors, and optimal CTV to PTV margins

Systematic errors (inter-patient), random errors (intra-patient) and the respective optimal CTV to PTV margins are shown in Table 2. The optimal margins for 3DUS were 5.1 mm in the lateral direction, 3.7 mm in the longitudinal direction and 5.5 mm in the vertical direction, while the optimal margins for bone-match in CBCT were 3.7 mm in the lateral direction, 6.1 mm in the longitudinal direction and 6.3 mm in the vertical direction. Obviously, the respective confidence intervals are largely intersecting in all cases. Hence, due to the small sample size (N = 6), no significant difference between any two methods could be detected.

**Table 2:** **Systematic and random error of 3DUS, CTV to PTV margin**

		**Lateral mm (95% CI)**	**Longitudinal mm (95% CI)**	**Vertical mm (95% CI)**
**Systematic errors**				
	3DUS	1.3 (0.8-3.3)	0.8 (0.5-2.1)	1.4 (0.9-3.6)
	CBCT (bone-match)	1.3 (0.8-3.1)	1.9 (1.2-4.6)	1.8 (1.4-5.4)
	Skin marks	1.6 (0.9-3.6)	2.2 (1.1-4.4)	2.3 (1.4-5.7)
**Random errors**				
	3DUS	2.5 (0.0-3.8)	2.3 (0.0-3.5)	2.7 (0.0-4.4)
	CBCT (bone-match)	0.7 (0.0-1.3)	2.0 (0.0-3.6)	2.7 (0.0-4.8)
	Skin marks	2.2 (0.0-3.8)	2.9 (0.0-4.7)	3.9 (0.0-5.7)
**Resulting optimal margins**				
	3DUS	5.1 (2.1-10.9)	3.7 (1.3-7.6)	5.5 (2.3-11.9)
	CBCT (bone-match)	3.7 (2.0-8.7)	6.1 (2.9-14.0)	6.3 (2.8-14.3)
	Skin marks	5.5 (2.4-12.2)	7.5 (3.4-16.8)	8.5 (3.6-18.1)

In units of mm; the systematic error is defined as the standard deviation of all individual mean errors; the random error is defined as the root of the average of all individual variances; the optimal margin is defined as 2.5 times the systematic error plus 0.7 times the random error.

## Discussion

Ultrasound-based guidance has improved the accuracy of prostate positioning for a definitive radiotherapy of prostate cancer [23,26,36,37]. Recently, using a calibration phantom, we have quantified the inherent technical limit of precision of the Clarity 3DUS system and found it to be less than 1 mm in all three directions [34,38]. In the present study, the Clarity system was investigated for intra-modality prostate positioning under clinical conditions. Our results showed that 3DUS is feasible for image-guided radiotherapy of prostate cancer. Using seed-match in CBCT as reference, 3DUS had systematic errors ranging from 0.8 to 1.4 mm and random errors ranging from 2.3 to 2.7 mm in all three directions. Compared to the other recently published data [26,32,37], our results are in quite the same range (Table 3). Notably, van der Meer et al. published their results of prostate alignments using the same 3DUS system and concluded that the overall accuracy of Clarity system is comparable to fiducial-based method [26].

**Table 3:** **Difference between setup errors measured in US vs seed-match in CBCT, compared to other published data, presented as mean ± SD**

**Reference**	**Lateral**	**Longitudinal**	**Vertical**
Bodda-Heggemann (2008) [37]	0.6 ± 1.7	0.9 ± 3.2	−1.7 ± 3.5
McNair (2006) [32]	−2.2 ± 3.7	3.2 ± 3.2	−3.3 ± 3.5
Van der Meer (2013) [26]	2.5 ± 4.0	0.6 ± 4.9	−2.3 ± 3.6
This paper	−0.2 ± 2.7	−1.9 ± 2.3	0.0 ± 3.0

In units of mm; N = 78 for 3DUS vs. seed-match in CBCT.

Further statistical analysis of our data showed that the discrepancies of setup errors between 3DUS and seed-match in CBCT were consistent with zero in the lateral and vertical directions, but significant in the longitudinal direction with mean ± SD of −1.9 ± 2.3 mm (Table 1). This significant difference is probably caused by a slight deviation in the calibration of 3DUS. One possible reason may be the absence of an image distortion correction when tissues are imaged which have a speed of sound different than the standard value employed by most US systems [39,40]. Theoretically, the intra-modality match may compensate such an aberration, provided that the same tissues are imaged in the simulation 3DUS and in the 3DUS before radiotherapy. However, in fact, the scanned tissues are mostly variable due to the difference of bladder filling and in the scan operations.

In this study, seed-match in CBCT was used as gold standard for the “real position” of prostate. However there are some limitations for its accuracy of prostate positioning. Namely, the implanted fiducials may change their positions in the prostate during a treatment series [24,41]. In the analysis of 6,111 measurements of inter-marker distances in 56 patients, Kupelian et al. showed that the average absolute variation ± SD of inter-marker distances were 1.01 ± 1.03 mm [41]. Beside the migration of markers in the prostate, the volume change of prostate, especially under anti-hormonal therapy, may also be responsible for the change of fiducial position. A detailed analysis of implanted markers showed an interesting time trend that the prostate volume increased slightly in the first week of the radiotherapy probably due to treatment-induced oedema, followed by a reduction of about 10% in the remaining 6 weeks of radiotherapy [24]. Thus, this point should also be taken into account in the interpretation of the different setup errors measured in 3DUS and seed-match in CBCT.

One important issue for the accuracy of US system is the dependence on the skill and experience of operators. The operators require more training in comparison to fiducial-based procedure in CBCT [36]. Fiandra et al. have carefully investigated this critical issue and shown that observers with less than one year experience had a significant larger deviation to the results of experts than the team with more than one year experience [42]. In our trial all the US scans were performed by two radiation oncologists and three radiation therapists, all of them received a training course by an expert trainer form Elekta company and had 12 months experience of clinical routine prior to the study presented here. All the 3DUS were retrospectively revised by the physicians (ML, FM).

Good quality of US imaging is a prerequisite for accurate guidance of prostate. For this purpose, a moderately filled bladder is needed for trans-abdominal US scan [26,28]. In this study, US-based prostate positioning was not performed for 16% of all fractions, partially due to insufficient bladder filling and thus limited imaging quality of US scans. This occurred mostly in the first one to two weeks of treatment, when the patients learned to manage a moderate bladder filling at the time of radiotherapy. Technically, an insufficient bladder filling can be corrected, if one lets the patients drink water and waits for the filling of bladder firstly, and then repeats the US scan. However this procedure will cost more time which was not consistently performed in the present study, since the final image guidance was based on seed-match in CBCT.

Another important issue for good quality of US imaging is adequate pressure of US probe on the skin. However in the case of trans-abdominal US, the pressure of probe on abdomen may change the position of prostate [26,43]. In BAT system the displacement of prostate was about 3 mm during correct usage while maximal displacement of 17 mm was reported if too much pressure was applied [43]. In Clarity system, van der Meer et al. have shown that relative low pressure (1 cm skin displacement on abdomen by probe) was sufficient for good image quality, so that the mean prostate displacement was −0.5 mm right, 0.7 mm posterior, and 0.0 mm superior [26]. In the present study, similar low pressure was preferably used for US scans, as long as good quality of US imaging was achieved. More importantly, since such prostate displacements occur both in simulation US and in US for prostate guidance before radiotherapy, and because an intra-modality match is performed, these displacements should have no impact on the accuracy of prostate positioning, as long as similar pressure is applied.

One limitation of the present investigation is the ignorance of rotational errors for prostate positioning. In the most bone-matches, the rotational errors were under 2°, while sometimes they exceed 4° in the seed-matches (data not shown). The 3DUS match is based directly upon the whole prostate organ and does not calculate rotational errors. Aubry et al. have investigated the rotational errors of prostate and shown that with a setup margin of 3 mm, only a negligible part of the prostate volume (average 0.09% and maximum 1.13%) will exceed this margin due to rotational errors [14]. In the absence of correction of rotational errors, such an additional margin should also be considered for the definition of setup margin.

## Conclusion

3DUS is feasible for image guidance for patients with prostate cancer and appears comparable to CBCT based image guidance in the retrospective study. While 3DUS offers some distinct advantages such as no need of invasive fiducial implantation and avoidance of extra radiation, its disadvantages include the operator dependence of the technique and dependence on sufficient bladder filling. Further study of 3DUS for image guidance in a large patient cohort is warranted.
